# Homocysteine metabolism in children and adolescents with epidermolysis bullosa

**DOI:** 10.1186/s12887-016-0714-7

**Published:** 2016-10-29

**Authors:** Rachele De Giuseppe, Greta Venturelli, Sophie Guez, Simona Salera, Claudia De Vita, Dario Consonni, Cinzia Dellanoce, Fabrizia Bamonti, Gabriella Chiarelli, Francesca Manzoni, Rita Maiavacca, Susanna Esposito

**Affiliations:** 1 Institute of Human Nutrition and Dietetics, Department of Public Health, Experimental and Forensic Medicine, University of Pavia, Pavia, Italy; 2 Internal Medicine and Metabolic Diseases; Fondazione IRCCS Ca’ Granda Ospedale Maggiore Policlinico, Milan, Italy; 3 Pediatric Highly Intensive Care Unit, University of Milan; Fondazione IRCCS Ca’ Granda Ospedale Maggiore Policlinico, Milan, Italy; 4 Department of Biomedical, Surgical and Dental Sciences, University of Milan, Milan, Italy; 5 Epidemiology Unit; Department of Preventive Medicine, Fondazione IRCCS Ca’ Granda Ospedale Maggiore Policlinico, Milan, Italy; 6 CNR Institute of Clinical Physiology, CardioThoracic and Vascular Department, Niguarda Ca’ Granda Hospital, Milan, Italy; 7 Laboratory of Clinical Chemistry and Microbiology, Fondazione IRCCS Cà Granda Ospedale Maggiore Policlinico, Milan, Italy

**Keywords:** Epidermolysis Bullosa, Homocysteine, Vitamin B_6_, Holotranscobalamin

## Abstract

**Background:**

Epidermolysis bullosa (EB) belongs to a family of rare heterogeneous, genetic disorders characterized by blistering of the skin and mucous membranes in response to minor mechanical trauma. The involvement of the oral mucosa and oesophagus stenosis is suggested to be responsible for severe nutritional deficiencies, but few studies have till now considered this aspect. This observational study aimed to evaluate homocysteine status in children and adolescents with EB by assessing total plasma homocysteine (tHcy) and metabolically related vitamins (B_6_, B_12_, folate) concentrations.

**Methods:**

Twenty EB patients (12 M; age range 0.5−19 years) were evaluated for: plasma tHcy, serum B_12_ and holotranscobalamin (HoloTC, the active fraction of B_12_), serum and erythrocyte folate (s-F and Ery-F, respectively), plasma B_6_ and serum high sensitive C-reactive-protein (hsCRP) levels. Clinical severity was also evaluated through the Birmingham Epidermolysis Bullosa Severity (BEBS) score. A sex and age well-matched population was also enrolled.

**Results:**

EB patients showed tHcy levels higher (*p* = 0.04) and B_6_ levels lower (*p* = 0.03) than controls. B_12_, HoloTC, s-F and ery-F concentrations did not differ between patients and controls. Multiple linear regression analysis showed that tHcy levels were independent of the metabolically related vitamins levels. In addition, serum hsCRP levels were higher in EB patients than in controls (*p* = 0.003) and correlated negatively with B_6_ concentrations (r = -0.6; *p* = 0.009). BEBS score correlated negatively with HoloTC (*p* = 0.022) and B_6_ (*p* = 0.005) levels and positively with age (*p* = 0.031) and hsCRP levels (*p* < 0.001).

**Conclusions:**

The assessment of tHcy and metabolically related vitamin levels describes an important aspect of EB patients’ nutritional *status* which can result essential for their long term care. Monitoring B_6_ levels in EB patients could be particularly important in order to prevent several complications associated with B_6_ deficiency and to avoid a B_6_ excess which sustains an inflammatory condition.

## Background

Epidermolysis bullosa (EB) belongs to a family of inherited autosomal (dominant or recessive) skin disorders. It is characterized by recurrent blistering formation of the skin and mucous membranes in response to minor mechanical trauma. The blisters easily rupture, and the resulting erosions and ulcerations are prone to infection [1, 2].

EB is classified into four main types, depending on the level of epidermal separation from the underlying basal lamina: EB Simplex (EBS), Junctional EB (JEB), Dystrophic EB (DEB), *Kindler* Syndrome (KS) [3]. Because of the involvement of the oral mucosa and oesophagus stenosis, EB patients, especially JEB and DEB types, are at risk of severe nutritional deficiencies, such as B vitamins group deficiencies (vitamin B_6_, vitamin B_9_ or folate, vitamin B_12_). This may be due to oral, oesophageal, and oropharyngeal problems (oral blistering and ulcerations, abnormal oesophageal motility, oesophageal strictures, dysphagia, and dental problems); digestion and absorption problems; anal erosions; fissures, and rectal strictures resulting in chronic constipation; loss of blood and protein through open skin blisters; and hyper-metabolism resulting in increased heat loss and protein turnover, especially in the setting of skin infections. These complications affect all infants, children, and adolescents with EB due to higher nutritional needs required for growth [2].

Hyperhomocysteinemia (HHcy) may be a result of one or more B vitamins group depletion and has been associated with several diseases, e.g. cardiovascular disease, Alzheimer disease and other dementias, peripheral neuropathy, renal failure and hypothyroidism [4–6]. Moreover, recent studies have demonstrated the involvement of homocysteine (Hcy) both in the enhancement of inflammatory activation and in autoimmunity triggering mechanisms, thus suggesting a possible role for Hcy not only in the development of cardiovascular disease but also in the pathogenesis of autoimmune diseases [7]. Since Hcy metabolism is catalysed by enzymes requiring B vitamins as cofactors, high total Hcy levels (tHcy) can indicate undernourishment due to a lack of metabolically related vitamins: in particular vitamin B_12,_ holotranscobalamin (HoloTC, the biologically active form of B_12_) [8] and/or folate, cofactors for the re-methylation pathway, and B_6_ for the transulfuration pathway [4].

Up to now few studies have taken into consideration EB patients’ nutritional deficiencies. To the best of our knowledge, this is the first study aimed at evaluating Hcy *status* by assessing tHcy and metabolically related vitamins levels in EB patients.

## Methods

### Subjects

Twenty consecutive EB children and adolescents (12 M/8 F; age range 0.5–19 years) were enrolled at the Pediatric Highly Intensive Care Unit of Fondazione IRCCS Cà Granda, Ospedale Maggiore Policlinico, Milan, Italy.

EB types were classified based on genetic, immunofluorescence mapping results, and clinical features, according with Fine JD. et al. [3].

Type analysis identified 6 cases of EBS (30 %), 1 case of JEB (5 %), 11 cases of DEB (55 %) and 2 cases of KS (10 %). Among DEB patients, 3 cases (27.3 %) were dominant dystrophic epidermolysis bullosa (DDEB) and 8 cases (72.7 %) were recessive dystrophic epidermolysis bullosa (RDEB).

In all patients, disease severity was also evaluated through the Birmingham Epidermolysis Bullosa Severity (BEBS) score. This score takes into account: area of damaged skin; involvement of nails, mouth, eyes, larynx and oesophagus; scarring of hands; skin cancer; chronic wounds; alopecia; nutritional compromise. Area was allocated 50 points, and the 10 other items 5 points each, giving a maximum score of 100 [9]. The BEBS score was performed by the paediatrician who regularly followed the patients (SG) at blood sample collection.

EB patients were compared with a healthy and well-matched age and gender control population (12 M/8 F; age range 1–19 years).

The study protocol was approved by the Ethics Committee of the Fondazione IRCCS Ca’ Granda Ospedale Maggiore Policlinico, Milan, Italy (Registration number: 2014–359), and conducted in accordance with the standards of Good Clinical Practice for trials of medicinal products in humans. The informed written consent of a parent or legal guardian was required for subjects aged <18, and the subjects aged ≥8 were asked to give their written assent. Patients’ parents and patients >8 years gave their written consent to data publication. Ethics Committee also approved data publication.

### Nutritional status

Children with severe EB tend to be short and underweight for their age. Nutritional status assessment was conducted by anthropometry. Patients and controls’ height and weight were measured under standard conditions by the same nurse, always using the same measuring equipment; Body Mass Index (BMI) was calculated. Weight, height and BMI for age were evaluated using Cacciari E. et al. growth curves [10].

### Biochemical analysis

Plasma tHcy and B_6_, serum folate (s-F), B_12_ and HoloTC, erythrocyte (ery-F) levels were measured in order to evaluate homocysteine metabolism. Serum high sensitive C reactive protein (hsCRP) was also assessed.

Blood samples were drawn in the morning, after an overnight fast. Two blood specimens from each patient were collected in light protected tubes, either with no additive (for serum B_12_, HoloTC, s-F and hsCRP) or containing ethylenediaminetetracetic acid (EDTA) to prevent coagulation, for ery-F, tHcy and B_6_ concentration assays. EDTA specimens were immediately put on ice and, after collecting whole blood aliquots for complete blood count and ery-F determination, were centrifuged within 30 min in order to obtain plasma samples for tHcy and B_6_ determination. Serum and plasma samples were frozen and stored at -80 ° C until analysed.

Plasma tHcy and serum B_12_ concentrations were determined by immunoenzymatic assay using the relevant commercial kits on AIA 600II analyser (Tosoh Bioscience, Tokyo, Japan), while HoloTC, s-F and ery-F by means of immunoenzymatic assay using the relevant commercial kits on Architect analyser i2000SR (Abbott Diagnostics, Abbott Park, IL, USA). Plasma B_6_ concentrations were evaluated by HPLC method using the relevant commercial kit (Chromsystems Instruments & Chemicals, Munich, Germany). HsCRP levels were assessed by immunoenzymatic assay using the relevant commercial kit on Modular P analyzer (Roche, Switzerland).

Because of the continuous physiological changes that occur throughout childhood, appropriately partitioned pediatric reference values are not readily available. For this reason, tHcy levels and metabolically related vitamins of EB patients were compared to those of a healthy population well-matched age and gender.

In addition, in our study, hyperhomocysteinemia and B_12_ deficiency were defined according to the reference intervals and *cut-off* values described by Bailey D. et al. in the Canadian Laboratory Initiative for Pediatric Reference Intervals (CALIPER) program [11] (Table 3), while B_6_, s-F and ery-F levels were compared to the data obtained from a meta-analysis which described biochemical vitamin-status data in different groups of the Spanish population [12] (Table 3). Particularly, assuming that B_6_, s-F and ery-F were normally distributed, we considered as reference interval the mean vitamins levels obtained in the Spanish Population, taking two standard deviations either side of the mean [12].

HoloTC levels were classified by using the relevant *cut-off* value for adult population [8] as any study has not yet performed in children and adolescent.

### Statistical analysis

Results, reported as the median value with InterQuartile Range (IQR; 25–75 percentiles), were analyzed using the Mann-Whitney test to assess any difference between EB patients and controls. In addition, because of the small number of JEB and KS subjects, only EBS and DEB groups’ Hcy metabolically related vitamins and hsCRP levels were compared.

Multiple linear regression analysis was used to find predictors of homocysteine values. Pearson’s coefficient was used to test the correlation between continuous variables. The statistical analysis was performed by using Stata 13 (StataCorp. 2013, Stata: Release 13 Statistical Software. College Station, TX: StataCorp LP).

## Results

Demographic characteristics and growth *outcomes* of patients and controls are reported in Table 1.

**Table 1:** Demographic characteristics and growth outcomes of patients and controls

	EB patients (*n* = 20)	Controls (*n* = 20)	*p* ^*^
Sex (M/F)	12/8	12/8	n.a.
age (years)	8.0;6.5-12.5	6.1;5.5-13	0.89
*Growth outcomes*			
Weight for age^a^	*n = 20*	*n = 20*	
< 3 p	35 % (7)	10 % (2)	0.13
3-10 p	10 % (2)	15 % (3)	0.99
10-25 p	15 % (3)	10 % (2)	0.99
25-50 p	10 % (2)	35 % (7)	0.13
50-75 p	20 % (4)	15 % (3)	0.99
75-90 p	5 % (1)	15 % (3)	0.60
90-97 p	5 % (1)	0 % (0)	0.99
Height for age^a^	*n = 20*	*n = 20*	
< 3 p	10 % (2)	10 % (2)	0.60
3-10 p	20 % (4)	20 % (4)	0.60
10-25 p	15 % (3)	10 % (2)	0.99
25-50 p	25 % (5)	20 % (4)	0.99
50-75 p	15 % (3)	5 % (1)	0.60
75-90 p	10 % (2)	15 % (3)	0.99
90-97 p	5 % (1)	20 % (4)	0.34
BMI (Kg/m^2^)^a^	*n = 20*	*n = 20*	
< 3 p	40 % (8)	20 % (4)	0.30
3-10 p	5 % (1)	15 % (3)	0.60
10-25 p	20 % (4)	10 % (2)	0.66
25-50 p	20 % (4)	20 % (4)	0.69
50-75 p	5 % (1)	25 % (5)	0.18
75-90 p	0 % (0)	10 % (2)	0.47
90-95 p	0 % (0)	0 % (0)	n.a.
95-97 p	5 % (1)	0 % (0)	0.99
> 97 p	5 % (1)	0 % (0)	0.99

Demographic characteristics are reported as median;IQR while growth *outcomes* are reported as percentage values and (number of subjects). ^*^ Mann-Whitney test for age and chi-square test for growth *outcomes*; *n.a* not applicable; ^**a**^ Cacciari E. et al*.* [10]

With regard to weight, 35 % (*n* = 7) and 10 % (*n* = 2) of patients and controls, respectively, were below the 3^rd^ percentile (*p* = 0.13); particularly, among these patients, 57 % were DEB (*n* = 4; RDEB), 29 % were EBS (*n* = 2) and 14 % were JEB (*n* = 1).

In regard to height, only 2 subjects were under the 3^rd^ percentile both in patients and in controls. Forty percent of patients (*n* = 8) and 20 % of controls (*n* = 4) had a BMI below the 3^rd^ percentile (*p* = 0.30); among these patients, 50 % were DEB (*n* = 4; RDEB), 37.5 % were EBS (*n* = 3) and 12.5 % were JEB (*n* = 1).

In addition, 1 DDEB patient (BEBS score = 2) showed both weight between the 90^th^-97^th^ percentile and BMI >95^th^ percentile; 1 EBS patient (BEBS score = 1) reported BMI > 95^th^ percentile.

Controls showed both weight and BMI under the 90^th^ percentile.

Among DEB patients, 5 of them (45.5 %) had oesophagus stenosis. Only one patient (JEB) was celiac while any other patients did not present clinical signs and symptoms of malabsorption.

BEBS score ranged from 2 to 67 points (Table 2).

**Table 2:** The Birmingham Epidermolysis Bullosa Severity (BEBS) score of EB patients

Patient	Sex (F: female; M: male)	Age (years)	EB classification	BEBS score
1	F	6	DDEB	2
2	M	8	KS	15
3	M	11	KS	17
4	M	19	RDEB	60
5	F	13	RDEB	50
6	M	10	EBS	8
7	F	7	RDEB	40
8	M	13	RDEB	65
9	M	3	RDEB	27
10	M	3	EBS	3
11	M	18	JEB	34
12	F	7	RDEB	32
13	M	8	EBS	1
14	F	3	EBS	25
15	F	13	EBS	17
16	M	0.5	EBS	3
17	M	7	DDEB	2
18	M	12	DDEB	1
19	F	7	RDEB	34
20	F	10	RDEB	67

*EBS* Epidermolysis Bullosa Simplex, *JEB* Junctional Epidermolysis Bullosa, *DDEB* Dominant Dystrophic Epidermolysis Bullosa, *RDEB* Recessive Dystrophic Epidermolysis Bullosa, *KS* Kindler Syndrome

Although we did not reported daily caloric and nutrient intake of our patients, 25 % of EB subjects (*n* = 4; 1 KS patient and 3 DEB patients) were taking calorically dense liquid supplements fortified with vitamins and minerals orally, containing vitamin B_12_, B_6_ and folic acid. Another 25 % of them (*n* = 4; 1 EBS patients, 3 DEB patients and 1 JEB patient) were supplemented only with vitamin B_12_. However the supplemented vitamin doses were less than the Italian Official Recommendation (IOR) vitamin intake [13].

Both EB patients and controls had normal renal functions (data not shown). The presence of microcytic anaemia was highlighted in 45 % of EB patients while controls’ hematologic parameters were within the relevant reference intervals (data not shown).

Biochemical parameters of patients and controls are listed in Table 3; results were reported as median value;interquartile range (median;IQR).

**Table 3:** Biochemical parameters of patients and controls

Analyte (reference interval or cut-off)	EB patients (*n* = 20)	Controls (*n* = 20)	*p* ^*^
tHcy^a^ (1 < 7 years; 2.7-7.6 μmol/L 7 < 12 years; 3.4 -8.4 μmol/L 12 < 15 years; M: 4.7-10.4 μmol/L; F: 4.1-10.4 μmol/L 15 < 19 years; M: 5.5-13.4 μmol/L; F: 4.9-11.9 μmol/L)	9.9;7.5-10.5 (55 %)	6.5;5.8-8.4 (15 %)	0.04 [0.02]
B_12_^a^ (1 < 9 years; 208.8- 1190 pmol/L 9 < 14 years; 185.9-830.2 pmol/L 14 < 17 years; 180.1-655.3 pmol/L 17 < 19 years; 149.8-598.5 pmol/L)	583.4;402.2-822.3 (0 %)	468.8;360.2-652.9 (5 %)	0.30 [1.00]
HoloTC (>40 pmol/L)	99.2;79.7-128 (10 %)	84.9;51.1-128 (10 %)	0.42 [0.60]
s-F^b^ (0-15 years; 11.6-33.2 nmol/L 15-65 years; 5.8-28.6 nmol/L)	11.1;8.4-21.3 (50 %)	13.1;11.1-15.7 (20 %)	0.64 [0.10]
ery-F^b^ (2-15 years; 409.9-1162 nmol/L)	843.3;591.0-1559.7 (10 %)	608.4;556.3-742.5 (10 %)	0.10 [0.60]
B_6_^b^ (2-5 years; 10.0-21.6 μg/L 9-13 years; 9.6-24.4 μg/L)	6.9;4.2-12.3 (70 %)	11.3;9.2-14.3 (25 %)	0.03 [0.01]
hsCRP (<0.5 mg/dL)	0.55;0.01-3.1 (50 %)	0.05;0.03-0.16 (0 %)	0.003 [0.00]

Data are reported as median;IQR. Percentage of altered values are reported in parentheses (%). ^*^Mann-Whitney test [chi-square test]

^a^ Bailey D. et al. [11]; ^b^ Ortega RM. et al. [12]. tHcy: total Homocysteine; B_12_: vitamin B_12_; HoloTC: Holotranscobalamin; s-F: serum Folate; ery-F: intraerythrocyte; B_6_: vitamin B_6_; hsCRP: high sensitive C Reactive Protein

**Fig. 1:**
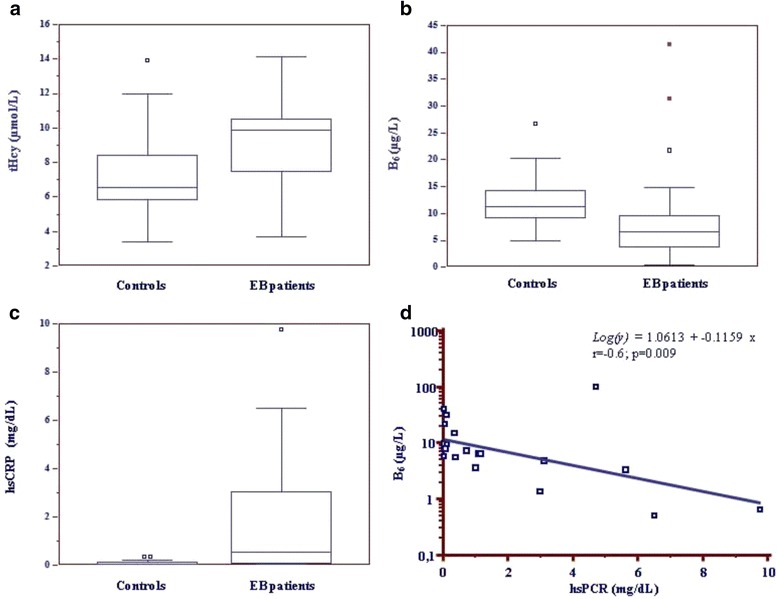
Biochemical parameters analysis in EB patients and controls. **a** Box-and-Whisker plot of tHcy levels between EB patients and controls (*p* = 0.04); **b** Box-and-Whisker plot of vitamin B_6_ levels between EB patients and controls (*p* = 0.03); **c** Box-and-Whisker plot of hsCRP levels between EB patients and controls (*p* = 0.003). Data are reported as median;Interquartile range (IQR); **d** Correlation between vitamin B_6_ and hsCRP levels in EB patients (*p* = 0.009)

Plasma tHcy levels were significantly higher in EB patients than in controls (9.9;7.5–10.5 μmol/L *vs* 6.5;5.8–8.4 μmol/L; *p* = 0.04) (Fig. 1a) and 55 % of patients showed tHcy levels above the *cut-off* value (10.4;10.1-10.9 μmol/L), according to CALIPER program’s reference interval [11]. Particularly, DEB patients showed significantly higher tHcy concentrations than controls (10.0;8.3-10.6 μmol/L *vs* 6.5;5.8-8.4 μmol/L; *p* = 0.03), while EBS patients’ tHcy levels did not differ significantly from controls’ levels (9.4;6.5-10.4 μmol/L *vs* 6.5;5.8-8.4 μmol/L; *p* = 0.25).

The evaluation of the Hcy metabolically related vitamins highlighted altered plasma B_6_ levels in 70 % of EB patients [12] and, interestingly, B_6_ levels were significantly lower in EB patients than in controls (6.9;4.2-12.3 μg/L *vs* 11.3;9.2-14.3 μg/L; *p* = 0.03) (Fig. 1b). Moreover, B_6_ levels were significantly lower both in patients with weight <3^rd^ percentile compared to the other patients (3.6;1.8-6.3 μg/L *vs* 9.1;6.9-18.3 μg/L; *p* = 0.03) and in patients with a BMI <3^rd^ percentile (4.6;2.3-6.2 μg/L *vs* 9.6;7.5-20.0 μg/L, *p* = 0.01) compared to the other patients.

S-F levels were altered in 50 % of EB subjects [12], and were significantly lower both in patients with weight <3^rd^ percentile compared to the other patients (8.6;6.1-10.4 nmol/L *vs* 13.8;10.4-22.4 nmol/L; *p* = 0.05) and in patients with BMI <3^rd^ percentile (7.6;5.8-9.9 nmol/L *vs* 15.9;11.0-23.3 nmol/L; *p* = 0.01) compared to the other patients. However median s-F concentrations between patients and controls did not show any difference (*p* = 0.64).

Serum B_12_ and HoloTC levels were normal both in most of EB patients and controls, according to Bailey D. et al. [11] and Bamonti F. et al studies [8], respectively.

No differences between patients and controls were observed in B_12_, HoloTC and ery-F levels.

However, interestingly, HoloTC concentrations were lower both in patients whose weight was under the 3^rd^ percentile, compared to the other EB patients (83.0;71.6-99.7 pmol/L *vs* 126.0;93.9-128.0 pmol/L; *p* = 0.09) and in patients with BMI <3^rd^ percentile (90.5;74.6-99.2 pmol/L *vs* 128.0;92.8-128.0 pmol/L; *p* = 0.08), compared to the other EB patients.

Despite the altered levels of B_6_ and s-F, multiple linear regression analysis showed that tHcy levels were independent of the metabolically related vitamins levels.

Serum hsCRP levels were significantly higher in EB patients than in controls (*p* = 0.003), altered in 50 % of patients, (Fig. 1c) and higher depending on the severity of EB type. In fact, hsCRP median levels were significantly higher both in DEB patients than controls (3.0;0.2-5.4 mg/dL *vs* 0.04;0.03-0.1 mg/dL; *p* = 0.000) and in DEB than in EBS patients (3.0;0.2-5.4 mg/dL *vs* 0.2;0.03-1.0, mg/dL; *p* = 0.04).

A negative correlation was found between hsCRP levels and B_6_ levels (r = -0.6; *p* = 0.009) (Fig. 1d).

Finally, BEBS score correlated negatively with HoloTC (r = -0.5; *p* = 0.022) and B_6_ (r = -0.6; *p* = 0.005) levels and positively with age (r = 0.5; *p* = 0.031) and hsCRP levels (r = 0.8; *p* < 0.001).

## Discussion

Hyperhomocysteinemia, a marker for folate, B_12_ and B_6_ deficiency, has been associated with enhancement of inflammatory activation and with autoimmunity triggering mechanisms [7]. Patients with EB suffer from acute and chronic malnutrition. In fact, in these patients, oesophagus stenosis and oral mucosa erosions are responsible for restricted ingestion, and protein-calorie malnutrition is worsened by losses from cutaneous blisters and by chronic inflammatory syndrome secondary to chronic skin infection [2]. Therefore, nutritional management is an essential aspect of the long term care of patients with EB because protein, vitamin and trace element intakes are essential for growth and wound healing and for the host resistance against bacterial infections.

Nowadays few studies have described vitamin status in EB. A previous study by Ingen-Housz-Oro S. et al. [14], described vitamin and trace metal status of 14 recessive DEB (RDEB) patients. The authors reported iron, vitamin D, C, B_6_, PP, zinc, and selenium deficiencies in 36–70 % of the patients, while vitamin B_1_, B_12_, B_2_, A, retinol binding protein and carnitine levels were within the relevant reference interval. For the first time, on the basis of these considerations, we evaluated, homocysteine status by assessing tHcy levels and metabolically related vitamins in 20 EB children and adolescents and compared them to those of a healthy population well-matched for age and gender. Our results showed tHcy levels above the *cut-off* value in more than 50 % of EB patients. Particularly, even if tHcy levels did no differ between EBS subjects and controls, DEB subjects’ tHcy levels were higher than in controls.

Analysing Hcy related vitamins our results concerning B_6_ were in agreement with previous findings [14, 15]; in fact, plasma B_6_ levels were under the lower limit of the reference interval in most of our patients [12] and lower in EB patients than in controls. As regards this vitamin deficiency, B_6_ levels in EB patients with weight under the 3^rd^ percentile, differed from B_6_ levels of patients with weight above the 10^th^ percentile. A plausible explanation of this deficiency was due to the EB patients’ undernourishment.

Shen J et al. [16] reported that plasma pyridoxal 5’-phosphate (PLP), the biologically active form of vitamin B_6_, was adversely associated with inflammatory markers, such as CRP, fibrinogen, and blood cell count. In addition, an inverse relationship between CRP and PLP has also been found among participants in the Framingham Heart Study and the National Health and Nutrition Examination Survey (NHANES) [17]. Current evidence suggests that the inverse association between plasma PLP and inflammation may be the result of this coenzyme used by the PLP-dependent enzymes for the kynurenine pathway of tryptophan degradation, for the metabolism of the immunomodulatory sphingolipids, ceramide and sphingosine 1-phosphate, and for serine hydroxymethylase for immune cell proliferation [17, 18]. According to the previous findings [17, 18], in the present study an inverse correlation was found between B_6_ levels and hsCRP in EB patients. In fact, our EB patients showed hsCRP levels higher than controls and, interestingly, higher in DEB than in EBS. No difference was found between EBS and controls. This was probably due to the extent of blistering and ulcerations, underlying a possible correlation between the severity disease and the inflammatory degree. Therefore, we hypothesized that B_6_ deficiency was not only a consequence of undernourishment but was also related to the systemic inflammation which characterized these patients.

Ery-F concentration is a reliable indicator of long-term folate *status* and general dietary intake, and s-F of more recent intake; a more comprehensive estimate of total folate *status* is generally obtained by assessing both parameters, as reported in this and in our previous studies [19–21]. In the present study, 50 % of the patients had altered s-F values due to undernourishment; in fact, in patients whose weight was under the 3^rd^ percentile, s-F levels were lower than in the other patients. However, s-Fol levels did not differ between the two groups, underlying only a mild deficiency in EB subjects. This was also confirmed by assessing ery-F levels which were within the relevant reference interval both in most patients and in controls and similar to those obtained by Ortega RM et al. [12].

The determination of cobalamin *status*, by measuring also HoloTC concentrations, represents an approach for diagnosing subtle cobalamin deficiency. HoloTC, the transcobalamin (TC)-cobalamin complex representing the biologically active form of the vitamin and consisting of 10 %–30 % of total serum B_12_, is recognized by ubiquitous specific membrane receptors and could have high diagnostic value as a marker of storage [8]. It has been demonstrated that HoloTC is a more sensitive marker of vitamin B_12_ *status* compared with total serum cobalamin [8], and it could be the earliest and most sensitive marker for vitamin B_12_ deficiency [8]. According to Ingen-Housz-Oro S. et al’*s* study [14], B_12_ levels were within the relevant reference interval in most patients and controls; this was probably due to the enterohepatic recycling preventing an early onset of cobalamin deficiency [22]. Although HoloTC levels did not differ significantly between the two groups, were altered in 10 % of patients and controls. Additionally, undernourished EB subjects weight <3^rd^ percentile) showed decreased HoloTC concentrations but vitamin B_12_ levels within the relevant reference interval when compared to the other well-nourished patients. Hence the importance of HoloTC determination, as an early marker of B_12_ deficiency, in order to monitor cobalamin *status.*

However, in the present study it was considered the HoloTC *cut-off* value of an adult population (*cut-off* value >40 pmol/L) [8] and this possible limitation could suggest that further studies are necessary in order to assess HoloTC levels in a larger paediatric population and create an appropriate *cut-off* value.

Finally, considering Hcy metabolism, despite the presence of B_6_ and s-F deficiency in some patients, the multiple linear regression analysis showed that tHcy levels did not depend on the metabolically related vitamins. A possible explanation is that it could be related to the limited number of studied patients; further studies are required to extend these preliminary results by expanding EB population.

A well-known fact is that even among EB patients within the same EB type, clinical symptoms could vary significantly as regards the severity of the disease. The nutritional *status* of these patients does not always correlate with their genetic diagnosis but is rather related to the clinical symptoms and could also be influenced by other socio-economic factors [23]. Therefore, in the present study, we scored disease severity for each patient using the BEBS [9] which might help to characterize patients with unexpectedly mild or severe disease, and contributing to genotype phenotype correlation [9]. As previously reported by other authors [9], BEBS score correlated positively with age, suggesting that changes in BEBS score reflect clinical observations, particularly disease progression with age. Additionally, BEBS score confirmed the B_6_ deficiency due to undernourishment and severe inflammation and the usefulness of HoloTC as an early marker of B_12_ deficiency.

The findings of our study are in agreement with the aforementioned consideration, particularly as regards BMI and B_6_ levels; in fact, in our population, 1 DDEB patient, despite the genetic diagnosis, showed good clinical symptoms (BEBS score = 2), BMI >95^th^ percentile and normal B_6_ levels. On the contrary, 3 EBS patients (BEBS score: 8; 17; 25) showed BMI <3^rd^ percentile and B_6_ levels under the lower limit of the reference interval [12].

## Conclusions

EB patients are at risk of severe nutritional deficiencies; to the best of our knowledge, this is the first study that took into consideration the possible nutritional deficiencies (B vitamins group) of young EB patients by evaluating Hcy *status.*

Most of EB patients showed tHcy levels above the *cut-off* value, probably due to the S-F and B_6_ levels deficiency. In fact, S-F levels decreased in half of the patients and even more so in the undernourished ones. However, by comparing EB patients’s-F levels with controls’, we noticed only a mild deficiency in EB patients. The consistent B_6_ deficiency was due not only to the undernourishment but also to the severe inflammation which in turn was underlined by an increase in EB patients’ hsCRP levels.

Finally, tHcy levels were not correlated significantly with metabolically related vitamins deficiency (particularly S-F levels and B_6_), because the limited number of studied cases.

In addition, we also reported the importance of HoloTC evaluation as an early marker of cobalamin deficiency.

In conclusion, monitoring tHcy and metabolically related vitamin levels enable us to describe the EB patients’ nutritional *status* could represent an essential aspect of their long term care. Monitoring B_6_ levels is particularly important in order to avoid both a number of complications associated with B_6_ deficiency and an excess of B_6_ which sustains an inflammatory condition.

## Abbreviations

BEBS: Birmingham Epidermolysis Bullosa Severity
BMI: Body mass index
DDEB: Dominant dystrophic epidermolysis bullosa
DEB: Dystrophic epidermolysis bullosa
EB: Epidermolysis bullosa
EBS: Epidermolysis bullosa simplex
EDTA: Ethylenediaminetetracetic acid
ery-F: erythrocyte
Hcy: Homocysteine
HHcy: Hyperhomocysteinemia
HoloTC: Holotranscobalamin
hsCRP: high sensitive C reactive protein
JEB: Junctional epidermolysis bullosa
KS: Kindler syndrome
RDEB: Recessive dystrophic epidermolysis bullosa
s-F: serum folate
tHcy: total Hcy levels

## References

[1] Shinkuma S, McMillan JR, Shimizu H (2011). Ultrastructure and molecular pathogenesis of epidermolysis bullosa. Clin Dermatol.

[2] Fedeles F, Murphy M, Rothe MJ, Grant-Kels JM (2010). Nutrition and bullous skin diseases. Clin Dermatol.

[3] Fine JD, Bruckner-Tuderman L, Eady RA, Bauer EA, Bauer JW, Has C (2014). Inherited epidermolysis bullosa: updated recommendations on diagnosis and classification. J Am Acad Dermatol.

[4] Malinowska J, Kolodziejczyk J, Olas B (2012). The disturbance of hemostasis induced by hyperhomocysteinemia; the role of antioxidants. Acta Biochim Pol.

[5] Seshadri S, Beiser A, Selhub J, Jacques PF, Rosenberg IH, D'Agostino RB (2002). Plasma homocysteine as a risk factor for dementia and Alzheimer's disease. N Engl J Med.

[6] Wu XQ, Ding J, Ge AY, Liu FF, Wang X, Fan W (2013). Acute phase homocysteine related to severity and outcome of atherothrombotic stroke. Eur J Intern Med.

[7] Lazzerini PE, Capecchi PL, Selvi E, Lorenzini S, Bisogno S, Galeazzi M, Laghi Pasini F (2007). Hyperhomocysteinemia, inflammation and autoimmunity. Autoimmun Rev.

[8] Bamonti F, Moscato GA, Novembrino C, Gregori D, Novi C, De Giuseppe R (2010). Determination of serum holotranscobalamin concentrations with the AxSYM ative B12 assay: cut-off point evaluation in the clinical laboratory. Clin Chem Lab Med.

[9] Moss C, Wong A, Davies P (2009). The Birmingham epidermolysis bullosa severity score: development and validation. Br J Dermatol.

[10] Cacciari E, Milani S, Balsamo A, Spada E, Bona G, Cavallo L (2006). Italian cross-sectional growth charts for height, weight and BMI (2 to 20 year). J Endocrinol Invest.

[11] Bailey D, Colantonio D, Kyriakopoulou L, Cohen AH, Chan MK, Armbruster D, Adeli K (2013). Marked biological variance in endocrine and biochemical markers in childhood: establishment of pediatric reference intervals using healthy community children from the CALIPER cohort. Clin Chem.

[12] Ortega RM, Mena MC, Faci M, Santana JF, Serra-Majem L (2001). Vitamin status in different groups of the Spanish population: a meta-analysis of national studies performed between 1990 and 1999. Public Health Nutr.

[13] Società Italiana di Nutrizione umana (SINU). LARN-Livelli di Assunzione di Riferimento di Nutrienti ed energia per la popolazione italiana-IV Revisione. Milano: Sics, Edizione; 2014.

[14] Ingen-Housz-Oro S, Blanchet-Bardon C, Vrillat M, Dubertret L (2004). Vitamin and trace metal levels in recessive dystrophic epidermolysis bullosa. J Eur Acad Dermatol Venereol.

[15] Fine JD, Tamura J, Johnson L (1989). Blood vitamin and trace metal levels in epydermolysis bullosa. Arch Dermatol.

[16] Shen J, Lai CQ, Mattei J (2010). Association of vitamin B-6 status with inflammation, oxidative stress, and chronic inflammatory conditions: the Boston Puerto Rican health study. Am J Clin Nutr.

[17] Ligi P, Ueland PM, Selhub J (2013). Mechanistic perspective on the relationship between pyridoxal 5'-phosphate and inflammation. Nutr Rev.

[18] Friso S, Jacques PF, Wilson PWF, Rosenberg IH, Selhub J (2001). Low circulating vitamin B_6_ is associated with elevation of the inflammation marker C-reactive protein independently of plasma homocysteine levels. Circulation.

[19] Bamonti F, Pellegatta M, Novembrino C, Vigna L, De Giuseppe R, de Liso F (2013). An encapsulated juice powder concentrate improves markers of pulmonary function and cardiovascular risk factors in heavy smokers. J Am Coll Nutr.

[20] Vener C, Novembrino C, Catena FB, Fracchiolla NS, Gianelli U, Savi F (2010). Oxidative stress is increased in primary and post-polycythemia vera myelofibrosis. Exp Hematol.

[21] De Vecchi AF, Bamonti-Catena F, Finazzi S, Campolo J, Taioli E, Novembrino C (2000). Homocysteine, vitamin B12, serum and erythrocyte folate in peritoneal dialysis and hemodialysis patients. Perit Dial Int.

[22] Rush EC, Katre P, Yajnik CS (2014). Vitamin B_12_: one carbon metabolism, fetal growth and programming for chronic disease. Eur J Clin Nutr.

[23] Fine JD (2010). Inherited epidermolysis bullosa: recent basic and clinical advances. Curr Opin Pediatr.

